# Self-feedbacks determine the sustainability of human interventions in eco-social complex systems: Impacts on biodiversity and ecosystem health

**DOI:** 10.1371/journal.pone.0176163

**Published:** 2017-04-28

**Authors:** Marco Ortiz, Richard Levins

**Affiliations:** 1 Instituto Antofagasta, Instituto de Ciencias Naturales AvH, Universidad de Antofagasta, Casilla, Chile; 2 Department of Global Health and Population, Harvard TH Chan School of Public Health, Harvard University, 665 Huntington Ave, Boston, MA, United States of America; University of Waterloo, CANADA

## Abstract

Several administrative polices have been implemented in order to reduce the negative impacts of fishing on natural ecosystems. Four eco-social models with different levels of complexity were constructed, which represent the seaweed harvest in central-northern Chile under two different regimes, Management and Exploitation Areas for Benthic Resources (MAEBRs) and Open Access Areas (OAAs). The dynamics of both regimes were analyzed using the following theoretical frameworks: (1) *Loop Analysis*, which allows the local stability or sustainability of the models and scenarios to be assessed; and (2) Hessian´s optimization procedure of a global fishery function (GFF) that represents each dynamics of each harvest. The results suggest that the current fishing dynamics in MAEBRs are not sustainable unless the market demand presents some type of control (i.e. taxes). Further, the results indicated that if the demand changes to a self-negative feedback (self-control) in MAEBRs, the stability is increased and, simultaneously, a relative maximum for the GFF is reached. Contrarily, the sustainability of the model/system representing the harvest (principally by cutting plants) in OAAs is not reached. The implementation of an “ecological” tax for intensive artisanal fisheries with low operational cost is proposed. The network analysis developed here is proposed as a general strategy for studying the effects of human interventions in marine coastal ecosystems under transient (short-term) dynamics.

## Introduction

The ever-increasing intensive human interventions in natural systems are making it progressively difficult to enact management policies within sustainable boundaries, especially when these processes occur in a complex eco-social context. The large-scale agriculture, forestry, and fishery dynamics are clear examples of human interventions in natural complex systems. Theoretical and practical approaches should integrate variables coming from the intersection among biology, ecology, and economy [1]. In order to mitigate further declines in the harvest, bio-diversity and health of marine ecosystems, several kinds of management tools have been implemented, such as Marine Protected Areas (MPAs), no-take zones [2, 3, 4], and Management and Exploitation Areas for Benthic Resources (MEABRs) [5], among others. Nevertheless, the success of these policies will depend on the awareness level of the fishers regarding the following two opposite forces: (1) their wish to protect the nature and environment, and (2) the pressure coming from the economic market (demand) in order to capitalize on nature and the environment. It is normally accepted that for economic systems, all resources provided from nature (as ecosystem services) are considered as potentially consumable goods.

Several studies have proposed that the fishers organizations in different locations around the world (including Chile) may be highly heterogeneous regarding their awareness for conservation, no-take areas, management and others [6, 7, 8, 9]; however, if the fishery is considered as a subsystem highly pressed by market demand, conservation efforts will depend in part on the price of the resources and profitability for industry. The analysis of marine populations exploited and the design of management policies are faced with at least the following two difficulties: (1) the commercial species are part of complex ecosystems, interacting with other species and creating networks. In this sense, any natural or human intervention will propagate effects though that network, being buffered along some pathways, amplified along others, and may even be inverted; and (2) our own interventions are not constant; we act on the system but also react to it, therefore our actions are in co-variation with the variables of the natural system. Thus, human interventions could introduce more uncertainties [10].

**Fig 1:**
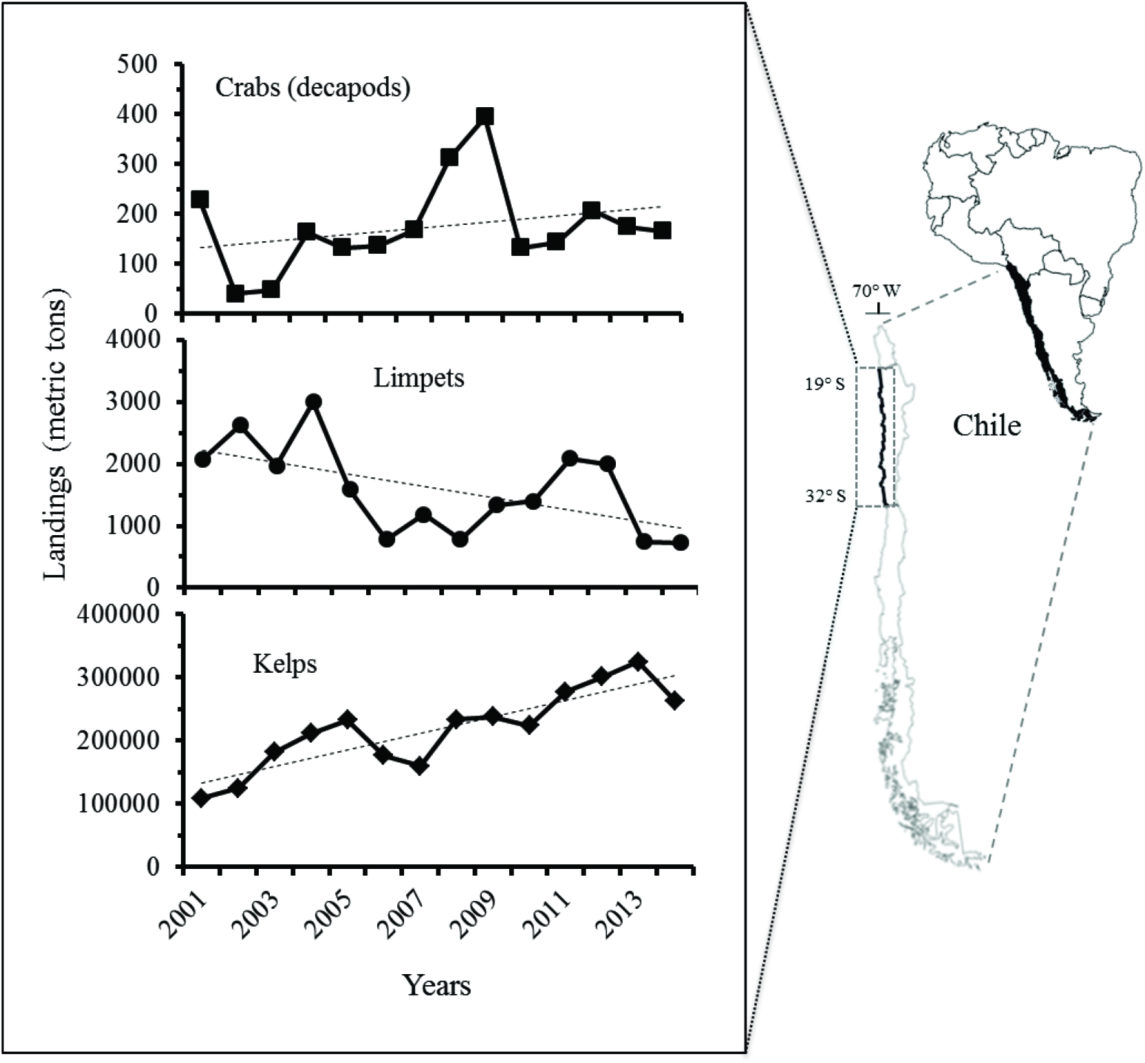
Historical landings (metric tons) of the interacting group of species analysed in the current study. The coastal region of central-north of Chile (SE Pacific) intervened by artisanal fishery is highlighted.

Over the last several years the scientific community has focused much attention on evaluating, quantifying, and predicting the changes that fisheries cause in the holistic properties of ecosystems [11, 12, 13, 14, 15]. In the last 13 years, the exploitation of kelp species *Lessonia* spp. (from intertidal habitats), *Lessonia trabeculata* and *Macrocystis pyrifera* (from subtidal areas) would show that the awareness for sustainability, protection and management could simply be an illusion. As shown in Fig 1, between the years 2001 and 2014, the total kelp landing increased from ~108,000 to ~260,000 tons in the central-north Chilean coast [16], representing at the end of year 2015 about 209 million USD for the industry [17]. The rampant increase of harvest is explained principally by the international pressure for raw material for alginic acid extraction and as a food source for abalone (*Haliotis discus hannoi*) farming [18, 19].

Different, not necessarily mutually exclusive, scientific strategies can be used to analyze, evaluate and attempt to predict the changes in the natural systems as a consequence of the concurrent seaweed fisheries and interacting species-groups along the central-north Chilean coast. These strategies include: (1) reducing the objects of study to their small components, assuming that the parts are more fundamental than the whole from which they are a part and supposing that the macroscopic or emergent properties of the whole ecosystems are an epiphenomenon of the parts; (2) creating a statistical “democracy” of factors, which assigns a relative weight and supposes that the factor that explains the greatest variance is the principal cause [20]; (3) quantitative simulation, supported by the capacity of computers and software to obtain numerical solutions, requiring fairly precise measurements of the variables, parameters, and exact equations; and (4) qualitative or semi-quantitative modeling, which allows integration of variables from different disciplines that do not need precise or quantitative equations [10, 21]. As a way to address the complexities related to this kind of exploitation, in the current work the semi-quantitative analysis is adopted; by doing so, we take advantage of the fact that we usually can know the qualitative direction of the effect of one variable on another even when we cannot describe their interaction precisely [10].

Due to the difficulty in carrying out replicated experiments to estimate the stability properties (considered as a measure for sustainability) and the propagation of direct and indirect effects of disturbances (such as fishing activities) on the community, ecosystem, or bio-geographical level [22, 23], in the current work we present four semi-quantitative models constructed to capture the main ecological (herbivory, predation, and commensalism) and eco-social relationships of this fishery. We use these models and scenarios to determine the local stability (as an indication of sustainability) of networks that describe MEABR and OAA subsystems using the *Loop Analysis* framework [21, 24], in turn, a multidimensional maximum or minimum for the harvest is estimated using the Hessian optimization analysis [25].

## Material and methods

### Ethic statement

No protected and endangered species were involved in this study. No vertebrate neither invertebrate species were collected in the present work. No sampling program was performed in situ because the models were built taken information from scientific literature. No specific permissions were required for this study area neither for the intellectual work.

### Loop Analysis and Hessian maximization theoretical frameworks

*Loop Analysis* is based on the correspondence among differential equations near equilibrium, Jacobian-Levins´s matrices, and their loop diagrams (digraphs). *Loop Analysis* [21, 24] is a useful procedure for estimating the local stability (as a sustainability measure) of systems and assessing the propagation of higher order effects as a response to external perturbations [21]. For more details of the modeling assumptions and basic equations see Supporting Information (S1 Appendix).

*Hessian Optimization Analysis* organizes all second partial derivatives of a multivariate function into a symmetric matrix. This multidimensional function integrates the overall behavior of different variables, and optimization conditions can be explored to find a relative maximum and minimum, and a saddle-point (ambiguous optimization) [25]. In this study, the multivariate global function was built additively due to the lack of knowledge of other types of complex interrelationships. For more details see Supporting Information (S1 Appendix). It is relevant to indicate that the *Hessian* analysis was qualitatively performed.

### Selection of boundaries, modeling structure and assumptions

Four semi-quantitative models and scenarios were built. The simplest version, model 1, represents the traditional population analysis, expanding the boundaries until the eco-social model 4 with three components exploited (connected by depredation and commensalism), the fishers and the demand. All models were constructed and analyzed simulating the harvest dynamics in Management and Exploitation Areas for Benthic Resources (MEABRs) and in the Open Access Areas (OAAs). Inside MEABRs, fishers and exploited species were simulated as self-damping (represented by a small circle), which means that fishing efforts are controlled by laws (limiting of number of fishers and boats, seasonal/reproductive bans, etc.) and the abundance of the species fluctuates between their carrying capacity (K) and K/2 (in the sense of a logistic equation), respectively. In contrast, in OAAs fishers and commercial species are self-enhanced (depicted by an arrow), representing a condition of illegal harvest (principally by cutting plants) with a reduced control as consequence of the vast coastal areas and an over-exploitation dynamic (with abundance < K/2) respectively. Even though the demand was simulated under self-enhancing dynamics since the market is dominated by positive feedbacks [26], it was also simulated under self-damping dynamics because this condition permitted a stable or sustainable scenario (SS) for the harvest of the *Concholepas concholepas* in northern Chile [10] to be reached. The positive feedbacks in economy result from the presence of non-linearities due to increasing returns or self-reinforcing mechanisms that induce multiple possible dynamic equilibriums [26]. Likewise, the positive feedbacks are known to lead frequently to instability, which would explain the highly changing artisanal fishing effort and target species. It is relevant to note that the self-damped dynamics (density-dependent growth rates) of commercial species indicates that they are slightly exploited; that is, their biomass is reduced between K and K/2 or they are simultaneously exploited and restocked. The over-exploitation, on the other hand, was simulated by self-enhanced dynamics (density-independent growth rates) [10]. For more details see Supporting Information (S2 Appendix).

#### Eco-social model 1

**Fig 2:**
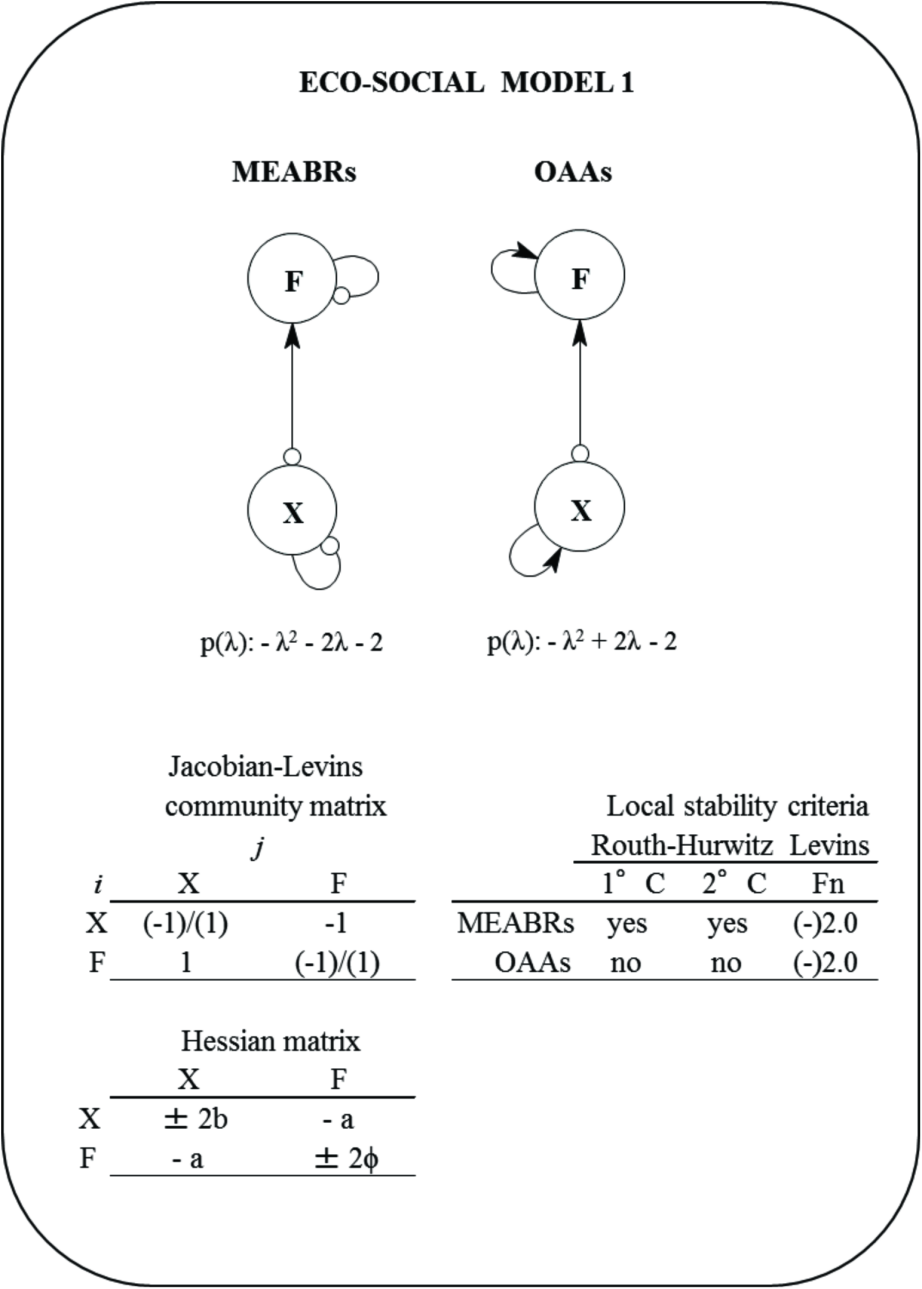
Eco-social Model 1 for Management and Exploitation Areas for Benthic Resources (MAEBRs) and Open Access Areas (OAAs). *F* = fishers, *X* = macroalgae, and p(λ) = characteristic polynomial. The community (Jacob-Levins) matrix with the semi-quantitative effect of *j* variable to *i* variable and the Hessian matrix are shown. The local stability measures of Routh-Hurwitz and Levins (*Fn*) criteria for MEABRs and OAAs scenarios are also summarized. First criterion (1°C) describes stability condition of the system, the second criterion (2°C) determine asymptotic or oscillation condition. The *Fn* is defined by holistic sustainability [25]. The assumptions included were changes in the self-dynamic (damped ‘-‘ [small circle] and/or enhanced ‘+’ [small arrow]) for fishers and macroalgae.

This model includes only two variables: fishers (*F*) and the group of seaweeds or kelp species (*X*). These variables are connected by a prey-predator relationship (Fig 2). The equations for each variable are the following:

$$\frac{dX}{dt}=r_{X}X-aXF\pm bX^{2}$$ (1)

$$\frac{dF}{dt}=q_{X}E_{X}X-wLF\pm \emptyset F^{2}$$ (2)

where, *r*_*x*_ = intrinsic growth rate of *X*, *a* = harvest transfer coefficient (from *F* on *X*), *b* = self-dynamics coefficient of *X*, *q*_*x*_ = catchability coefficient of fishing on *X*, *E*_*x*_ = fishing effort on *X*, *w* = law pressure coefficient on *F*, *L* is fishery laws, *Φ* = self-feedback coefficient of *F*. The positive and negative self-dynamics for *X*, *F* and *D* are summarized in Supporting Information (S2 Appendix).

The multidimensional global fishery function (GFF) to be used by Hessian optimization analysis was built up additively integrating *dX/dt* and *dF/dt* as follows:

$$GFF=\frac{dX}{dt}+\frac{dF}{dt}=r_{X}X-aXF\pm bX^{2}+q_{X}E_{X}X-wLF\pm \emptyset F^{2}$$ (3)

The Jacobian-Levins and Hessian matrices are shown in Fig 2. The same procedures were repeated for the other models.

#### Eco-social model 2

**Fig 3:**
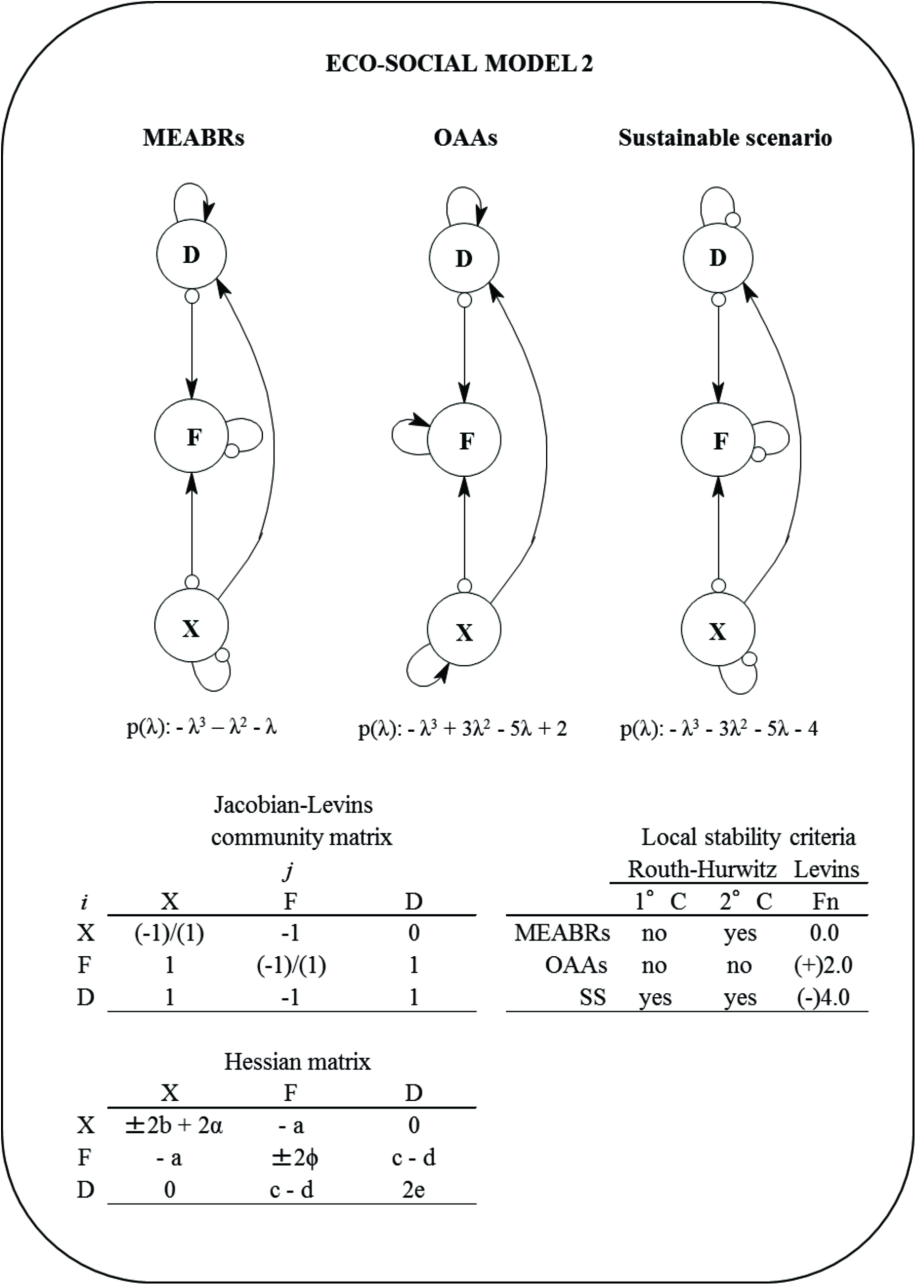
Eco-social Model 2 for Management and Exploitation Areas for Benthic Resources (MAEBRs), Open Access Areas (OAAs), and sustainable scenario (SS). *D* = demand, *F* = fishers, *X* = macroalgae, and p(λ) = characteristic polynomial. The community (Jacob-Levins) matrix with the semi-quantitative effect of *j* variable to *i* variable and the Hessian matrix are shown. The local stability measures of Routh-Hurwitz and Levins (*Fn*) criteria for MEABRs, OAAs and sustainable scenarios are also summarized. First criterion (1°C) describes stability condition of the system, the second criterion (2°C) determine asymptotic or oscillation condition. The *Fn* is defined by holistic sustainability [25].The assumptions included were changes in the self-dynamic (damped ‘-‘ [small circle] and/or enhanced ‘+’ [small arrow]) for fishers and macroalgae.

This model is an extension of the first configuration. It maintains the initial conditions of the previous model and includes one new variable, the demand (*D*). Demand has a positive influence on fishermen (*F*), but the fishermen have a negative effect on demand (Fig 3). The equations for each variable are:

$$\frac{dX}{dt}=r_{X}X-aXF\pm bX^{2}$$ (4)

$$\frac{dF}{dt}=q_{X}E_{X}X+cDF-wLF\pm \emptyset F^{2}$$ (5)

$$\frac{dD}{dt}=\alpha X^{2}-dFD+eD^{2}$$ (6)

The components of each equation are similar to the previous model, including *c* = coefficient of effect from demand to fishermen, *α* = coefficient effect of *X* on demand, *d* = coefficient of effect from fishermen to demand, and *e* = self-feedback coefficient of demand. The positive and negative self-dynamics for *X*, *F* and *D* are summarized in Supporting Information (S2 Appendix). Fig 3 shows the Jacobian-Levins and Hessian matrices.

The multivariate global fishery additive function (GFF) for this model is given by:

$$GFF=r_{X}X-aXF\pm bX^{2}+q_{X}E_{x}X+cDF-wLF\pm \emptyset F^{2}+\alpha X^{2}-dFD+eD^{2}$$ (7)

#### Eco-social model 3

**Fig 4:**
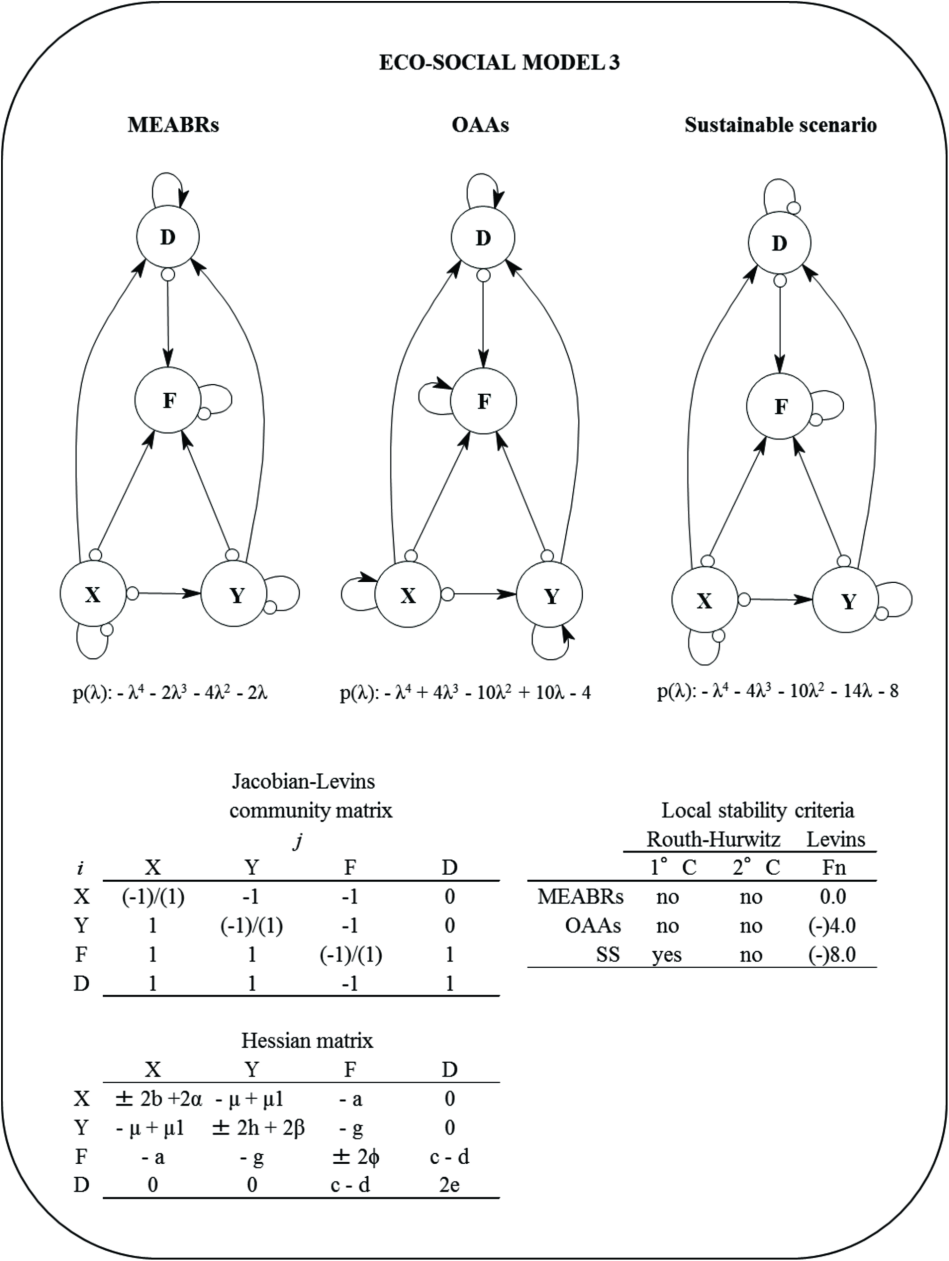
Eco-social Model 3 for Management and Exploitation Areas for Benthic Resources (MAEBRs), Open Access Areas (OAAs), and sustainable scenario (SS). *D* = demand, *F* = fishers, *X* = macroalgae, *Y* = herbivores, and p(λ) = characteristic polynomial. The community (Jacob-Levins) matrix with the semi-quantitative effect of *j* variable to *i* variable and the Hessian matrix are shown. The local stability measures of Routh-Hurwitz and Levins (*Fn*) criteria for MEABRs, OAAs and sustainable scenarios are also summarized. First criterion (1°C) describes stability condition of the system, the second criterion (2°C) determine asymptotic or oscillation condition. The *Fn* is defined by holistic sustainability [25].The assumptions included were changes in the self-dynamic (damped ‘-‘ [small circle] and/or enhanced ‘+’ [small arrow]) for fishers, macroalgae and herbivores.

This abstraction includes one new variable corresponding to the keyhole limpet group (*Y*), which is connected with the seaweeds by a plant-herbivore relationship (Fig 4). The equation for each variable corresponds to:

$$\frac{dX}{dt}=r_{X}X-aXF-\mu XY\pm bX^{2}$$ (8)

$$\frac{dY}{dt}=\mu_{1}YX-gYF\pm hY^{2}$$ (9)

$$\frac{dF}{dt}=q_{X}E_{X}X+q_{Y}E_{Y}Y+cDF-wLF\pm \emptyset F^{2}$$ (10)

$$\frac{dD}{dt}=\alpha X^{2}+\beta Y^{2}-dFD+eD^{2}$$ (11)

where, *μ* = feeding transfer coefficient from *Y* to *X*, *μ*_*1*_ = feeding transfer coefficient from *X* to *Y*, *g* = harvest transfer coefficient (from *F* to *Y*), *h* = self-feedback coefficient of *Y*, *q*_*Y*_ = catchability coefficient of fishing on *Y*, *E*_*Y*_ = fishing effort on *Y*, *β* = coefficient effect of *Y* on demand. The positive and negative self-dynamics for *X*, *Y*, *F* and *D* are summarized in Supporting Information (S2 Appendix). The Jacobian-Levins and Hessian matrices are depicted in Fig 4.

The multidimensional global fishery function (GFF) for this model was:

$$GFF=r_{X}X-aXF-\mu XY\pm bX^{2}+\mu_{1}YX-gYF\pm hY^{2}+q_{X}E_{X}X+q_{Y}E_{Y}Y+cDF-wLF\pm \emptyset F^{2}+\alpha X^{2}+\beta Y^{2}-dFD+eD^{2}$$ (12)

#### Eco-social model 4

**Fig 5:**
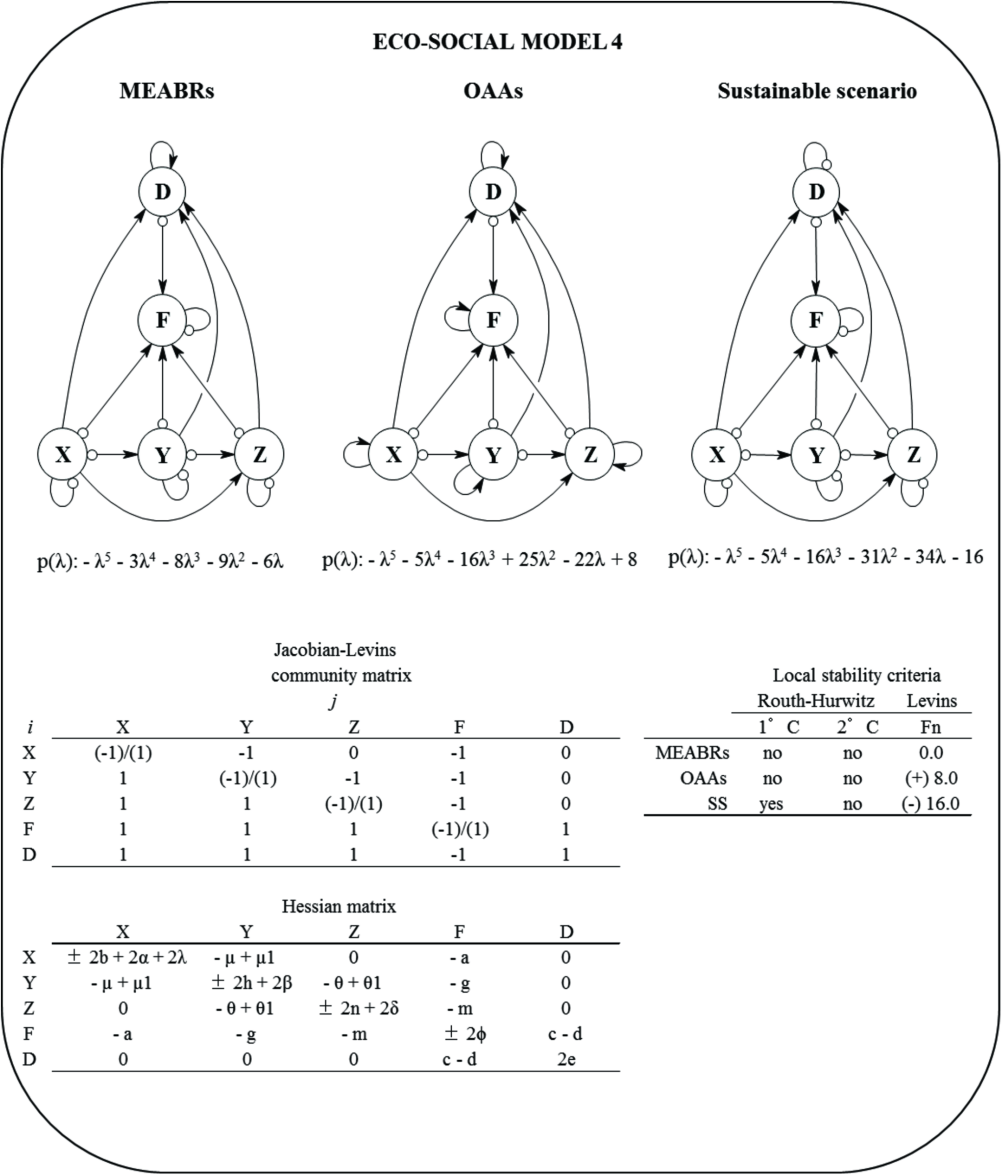
Eco-social Model 4 for Management and Exploitation Areas for Benthic Resources (MAEBRs), Open Access Areas (OAAs), and sustainable scenario (SS). *D* = demand, *F* = fishers, *X* = macroalgae, *Y* = herbivores, *Z* = carnivores, and p(λ) = characteristic polynomial. The community (Jacob-Levins) matrix with the semi-quantitative effect of *j* variable to *i* variable and the Hessian matrix are shown. The local stability measures of Routh-Hurwitz and Levins (*Fn*) criteria for MEABRs, OAAs and sustainable scenarios are also summarized. First criterion (1°C) describes stability condition of the system, the second criterion (2°C) determine asymptotic or oscillation condition. The *Fn* is defined by holistic sustainability [25].The assumptions included were changes in the self-dynamic (damped ‘-‘ [small circle] and/or enhanced ‘+’ [small arrow]) for fishers, macroalgae, herbivores and carnivores.

Finally, this model conserves all relationships and dynamics considered in the previous models and incorporates one new variable: the crab group (*Z*) connected as a predator upon keyhole limpets and being positively impacted by the seaweeds (commensalism), since these algae offer refuge and protection for recruits and juveniles (Fig 5). The equation for each variable is:

$$\frac{dX}{dt}=r_{X}X-aXF-\mu XY\pm bX^{2}$$ (13)

$$\frac{dY}{dt}=\mu_{1}YX-\theta YZ-gYF\pm hY^{2}$$ (14)

$$\frac{dZ}{dt}=\theta_{1}ZY+\lambda X^{2}-mZF\pm nZ^{2}$$ (15)

$$\frac{dF}{dt}=q_{X}E_{X}X+q_{Y}E_{Y}Y+q_{Z}E_{Z}Z+cDF-wLF\pm \emptyset F^{2}$$ (16)

$$\frac{dD}{dt}=\alpha X^{2}+\beta Y^{2}+\delta Z^{2}-dFD+eD^{2}$$ (17)

where, *θ* = feeding transfer coefficient from *Z* to *Y*, *θ*_*1*_ = feeding transfer coefficient from *Y* to *Z*, *m* = harvest transfer coefficient (from *F* to *Z*), *n* = self-feedback coefficient of *Z*, *λ* = protection coefficient from *X* to *Z* (commensalism), *q*_*Z*_ = catchability constant of fishing on *Z*, *E*_*Z*_ = fishing effort on *Z*, *δ* = coefficient effect of *Z* on demand. The positive and negative self-dynamics for *X*, *Y*, *F* and *D* are summarized in Supporting Information (S2 Appendix). Fig 5 shows the Jacobian-Levins and Hessian matrices.

The additive multivariate global fishery function (GFF) for this model is:

$$GFF=r_{X}X-aXF-\mu XY\pm bX^{2}+\mu_{1}YX-\theta YZ-gYF\pm hY^{2}+\theta_{1}ZY+\lambda X^{2}-mZF\pm nZ^{2}+q_{X}E_{X}X+q_{Y}E_{Y}Y+q_{Z}E_{Z}Z+cDF-wLF\pm \emptyset F^{2}+\alpha X^{2}+\beta Y^{2}+\delta Z^{2}-dFD+eD^{2}$$ (18)

## Results

The simplest eco-social abstraction represented by Model 1 (Fig 2) shows that the exploitation of the seaweed group (*X*) would be sustainable (under three stability criteria) only if the fishermen (*F*) and seaweeds (*X*) are self-damped (MEABR scenario). Likewise, for this same scenario, a relative maximum for the multidimensional global fishery function (GFF) was found using the Hessian analysis (Table 1). Contrarily, if *X* and *F* are self-enhancing, the system would not be stable and *GFF* would be relatively minimized (OAA scenario) (Table 1). Regarding Model 2, which includes the demand (*D*) (Fig 3), the exploitation could be sustainable (under three stability criteria) only if seaweeds, fishers and demand are self-damped (sustainable scenario, SS), and a relative maximum for the GFF would be reached (Table 1). In contrast, if *D*, *F* and *X* present a self-enhancing dynamic, the local stability is not reached and a relative minimum for GFF could be estimated (OAA scenario) (Table 1). In the case of the MEABR scenario, the model system would be asymptotically oscillatory stable and a saddle-point (ambiguous optimization) for GFF is determined (Table 1).

**Table 1:** Qualitative Hessian optimization outcomes for the eco-social models 1 & 2 and scenarios analysed in the current study. *D* = the determinant of Hessian matrix, and *i* = number of variables. For more details see Supporting Information (S3 Appendix).

Model 1			Model 2		
Determinant of	MEABRs	OAAs	MEABRs	OAAs	Sustainable
Hessian matrixes					scenario
A. Qualitative analysis					
D_i = 1_	(-) 2b, < 0	(+) 2b, > 0	(-) P, < 0	(+) L, > 0	(-) P, < 0
D_i = 2_	(+) 4bϕ—a^2^, > 0	(+) 4bϕ—a^2^, > 0	(+) 2Pϕ—a^2^, > 0	(+) 2Lϕ—a^2^, > 0	(+) 2Lϕ—a^2^, > 0
D_i = 3_	-	-	(+) 4Pϕe - 2a^2^e, > 0	(+) 4Lϕe - 2a^2^e, > 0	(-) 4Lϕe + 2a^2^e, < 0
D_i = 4_	-	-	-	-	-
D_i = 5_	-	-	-	-	-
Hessian results for *GFF*	Rel. Max.	Rel. Min.	Saddle-point	Rel. Min.	Rel. Max.

Fig 4 shows the outcomes of local stability for Model 3. This eco-social system is damped oscillatory stable only if the demand, fishers, seaweeds and keyhole limpets are self-damped (sustainable scenario) (Table 2). Contrarily, if these variables describe self-positive dynamics, the system will not achieve the necessary stability (OAA scenario) (Table 2). As in the previous model, a relative maximum and minimum for GFF is found for all variables under self-damping (sustainable scenario) and self-enhancing dynamics (OAA scenario), respectively. The MEABR eco-social scenario would not be stable and a saddle-point for GFF is reached (Table 2).

**Table 2:** Qualitative Hessian optimization outcomes for the eco-social model 3 and scenarios analysed in the current study. *D* = the determinant of Hessian matrix, and *i* = number of variables. For more details see Supporting Information (S3 Appendix).

Model 3			
Determinant of	MEABRs	OAAs	Sustainable scenario
Hessian matrixes			
A. Qualitative analysis			
D_i = 1_	(-) P, < 0	(+) L, > 0	(-) P, < 0
D_i = 2_	(+) PT, > 0	(+) LS, > 0	(+) PT, > 0
D_i = 3_	(-) PTϕ + 2Pg^2^e + 2Ta^2^e, < 0	(+) 2LSϕ—Lg2—Sa2, > 0	(-) 2PTϕ + Pg^2^ + Ta^2^, < 0
D_i = 4_	(-) 4PTϕe + 2Pg^2^e + 2Ta^2^e, < 0	(+) 4LSϕe -2Lg^2^e - 2Sa^2^e, > 0	(+) 4PTϕe - 2Pg^2^e - 2Ta^2^e, > 0
D_i = 5_	-	-	-
Hessian results for *GFF*	Saddle-point	Rel. Min.	Rel. Max.

The stability results obtained from Model 4 are summarized in Fig 5. As for the previous models, the relative maximum for GFF is only possible to estimate if all variables are self-damping (Table 3); that is, coinciding with a damped oscillatory stable sustainable scenario (SS). Conversely, a locally unstable and/or unsustainable relative minimum for GFF would be determined only if *X*, *Y*, *Z*, *F* and *D* are self-enhancing (OAA scenario). In this model, the MEABR scenario would be locally unstable or unsustainable and an ambiguous optimization for GFF is determined (Table 3).

**Table 3:** Qualitative Hessian optimization outcomes for the eco-social model 4 and scenarios analysed in the current study. *D* = the determinant of Hessian matrix, and *i* = number of variables. For more details see Supporting Information (S3 Appendix).

Model 4			
Determinant of	MEABRs	OAAs	Sustainable scenario
Hessian matrixes			
A. Qualitative analysis			
D_i = 1_	(-) P, < 0	(+) L, > 0	(-) P, < 0
D_i = 2_	(+) PT, < 0	(+) LS, > 0	(+) PT, > 0
D_i = 3_	(-) PTU, < 0	(+) LSV, < 0	(-) PTU, < 0
D_i = 4_	(+) 2PTUϕ—PTm2—PUg2—TUa2, > 0	(+) 2LSVϕ—LSm2—LVg2—SVa2, > 0	(+) PTUϕ—PTm^2^—PUg^2^—TUa^2^, > 0
D_i = 5_	(+) 4PTUϕe - 2PTm^2^e - 2PUg^2^e - 2TUa^2^e, > 0	(+) 4LSVϕe - 2LSm^2^e - 2LVg^2^e - 2SVa^2^e, > 0	(-) 4PTUϕe + 2PTm^2^e + 2PUg^2^e + 2TUa^2^e, < 0
Hessian results for *GFF*	Saddle-point	Rel. Max.	Rel. Max.

It is relevant to indicate that although the optimization outcomes obtained were independent of the order of variables into the Hessian matrices, two special conditions were relevant to estimate a relative maximum or minimum for GFF: (1) the strength of self-feedbacks of variables must be higher than the other coefficients in the system; and (2) the protection function offered by macroalgae (*λ*) to the crabs should be lower than the self-feedback of the macroalgae, since an ambiguous outcome (saddle-point) is obtained. For more details see Supporting Information (S3 Appendix).

## Discussion

Four models and scenarios for eco-social relationships that describe the exploitation of seaweeds, keyhole limpets and crabs along the central and northern Chile were constructed, each with different levels of complexity, and the models were analyzed by *Loop Analysis* [1, 21, 24] and Hessian optimization analysis [25]. Although all four models are a partial representation of the variables and interactions underlying the ecological and social subsystem studied, it is relevant to mention that this limitation is applicable to any kind of model and is independent of its degree of complexity [27, 28]. The models described herein would have at least the following simplifications: (1) the ecological complexity was reduced to just three interacting variables (seaweeds, keyhole limpets and crabs), ignoring the other species inhabiting the benthic and pelagic environment; (2) the community was considered in a moving equilibrium; (3) only two variables (demand and fishers) from the socio-economic subsystem were included in the models; and (4) the strength of self-feedbacks was a necessary condition for estimation of maximum and minimum of GFF, independent of the order of variables in the Hessian matrices. In spite of these limitations, we claim that the outcomes reached are sufficiently robust given the agreement among different models (independent of the level of complexity), thereby permitting us to assess the effects of the intensive exploitation of seaweeds in central and northern Chile. For our purposes, equilibrium was considered as a form of coordinated motion (especially in a transient or short-term dynamic) that includes a set of variables of the system from which the system will not move unless perturbed.

The outcomes obtained using Model 1 represent the simplest and commonest approach for trying to conserve and manage the natural bio-resources, which may be considered similar to those results obtained using the logistic equation for exploited populations. Models 2, 3 and 4 showed, however, that the scenario describing the current MEABR dynamics -under a holistic view- would be unsustainable (even with abundance of target species are oscillating between K and K/2) without a maximum or a minimum for GFF unless the demand (D) is controlled. Therefore, the model results would contradict the idea that the current management activities inside MEABRs can be considered as a sustainable tool promoting the conservation of species, especially if the MEABRs represent less < 10% of the Chilean coast.

The landings of seaweeds observed between the years 2001 and 2014 along the Chilean coast (Fig 1) would represent clearly that this resource has been over-exploited in OAAs, especially if the legal harvest consists only of collection of plants on the beaches that were naturally detached from the hard bottoms by water movement, wave impact and bottom currents. Therefore, the drastic increase of landings experienced in the last years is likely a consequence of illegal extractive harvest by cutting plants in intertidal and subtidal environments [19, Ortiz *personal observation*]. This agrees with our models, which show that, independent of the complexity level, the harvest dynamics of seaweeds observed the last 13 years, principally in OAAs along the Chilean coast, would cause an unstable or unsustainable relative minimum for this fishery. A similar unsustainable dynamic with over-exploitation may be also suggested for the other interacting species. Our assumption that over-exploited seaweeds are described by self-positive dynamics (density-independent growth rates) are partially corroborated by a field study recently carried out along a fraction of the coast of central-north Chile (from 26° to 31° S), which has showed that the density of adult and recruits of *L*. *nigrescens* in OAAs were significantly lower and higher respectively (changing the size structure), compared to inside MAEBRs [29].

In the early 1990s, before the drastic increase in seaweed landings, it had already been proposed that in order to design and apply successful management strategies for the harvest of these kelp species should integrate bio-ecological and social aspects such as: (1) the pressure of the international market (demand); (2) unemployment indexes of coastal workers (as occasional fishers); (3) monitoring and control of management recommendations; and (4) education level of fishers [30]. Since that time, several studies have analyzed the behavior of the Chilean kelp fishery, and have suggested what biological and ecological factors should be considered in a general management plan for *L*. *nigrescens*, which is the principal exploited kelp species from intertidal habitats [18, 31, 32]. Even though these contributions described relevant key aspects for kelp species population dynamics, the existing studies have not incorporated, explicitly, the positive influence of the demand coming from the market subsystem on fishers. Historically, the control policies for any fishery have only considered aspects concerning the fishers such as effort level, catch quotas, number of fishers, the number of intermediated buyers, and the implementation of several types of bans. Nevertheless, a sustainability approach with a control mechanism impacting the demand has not been implemented to date. Our results showed that holistic sustainable scenarios with a maximized GFF were obtained if fishing operates when the abundance of commercial species fluctuates between K and higher than K/2; therefore, re-stocking programs and local aquaculture operations should be strongly encouraged. It is interesting to mention that between K and K/2 lie the fishing efforts corresponding to the maximum economic yield (E_MEY_), which is lower than the effort at maximum sustainable yield (E_MSY_) [33, 34].

In the economic context, the demand is dominated by self-positive feedbacks [26]; however, this dynamic promotes unstable states for MEABRs and OAAs eco-social complex networks. Self-damped dynamics for fishers alone does not ensure the necessary local stability of harvest since it remains mainly sensitive to the self-enhanced dynamic coming from the economy (demand). Based on these outcomes, we conclude that the success of management instruments like MEABRs, Marine Protected Areas (MPAs), and no-take zones would not only depend on the awareness level of the fishers, but fundamentally on the demand. In this sense, Bodini et al. [35] concluded also that stability and sustainability of management strategies would depend on the prevalence of self-negative feedbacks over positive ones. Therefore, while the authorities have not considered implementing a policy addressed at controlling the demand, for instance by taxing, the sustainability of this fishery-as a whole-, is unlikely to be reached. Likewise, it is important also indicate that, in practical terms, any control mechanisms for harvest and fishers could be successfully monitored only inside MEABRs, whereas the vast extensions of OAAs would remain over-exploited [29], reducing the possibility of exerting effective fishing control by authorities [36]. Similar conclusions were drawn after modeling the harvest of *C*. *concholepas*, another important marine resource that inhabits benthic environments along the Chilean coast [10]

**Fig 6:**
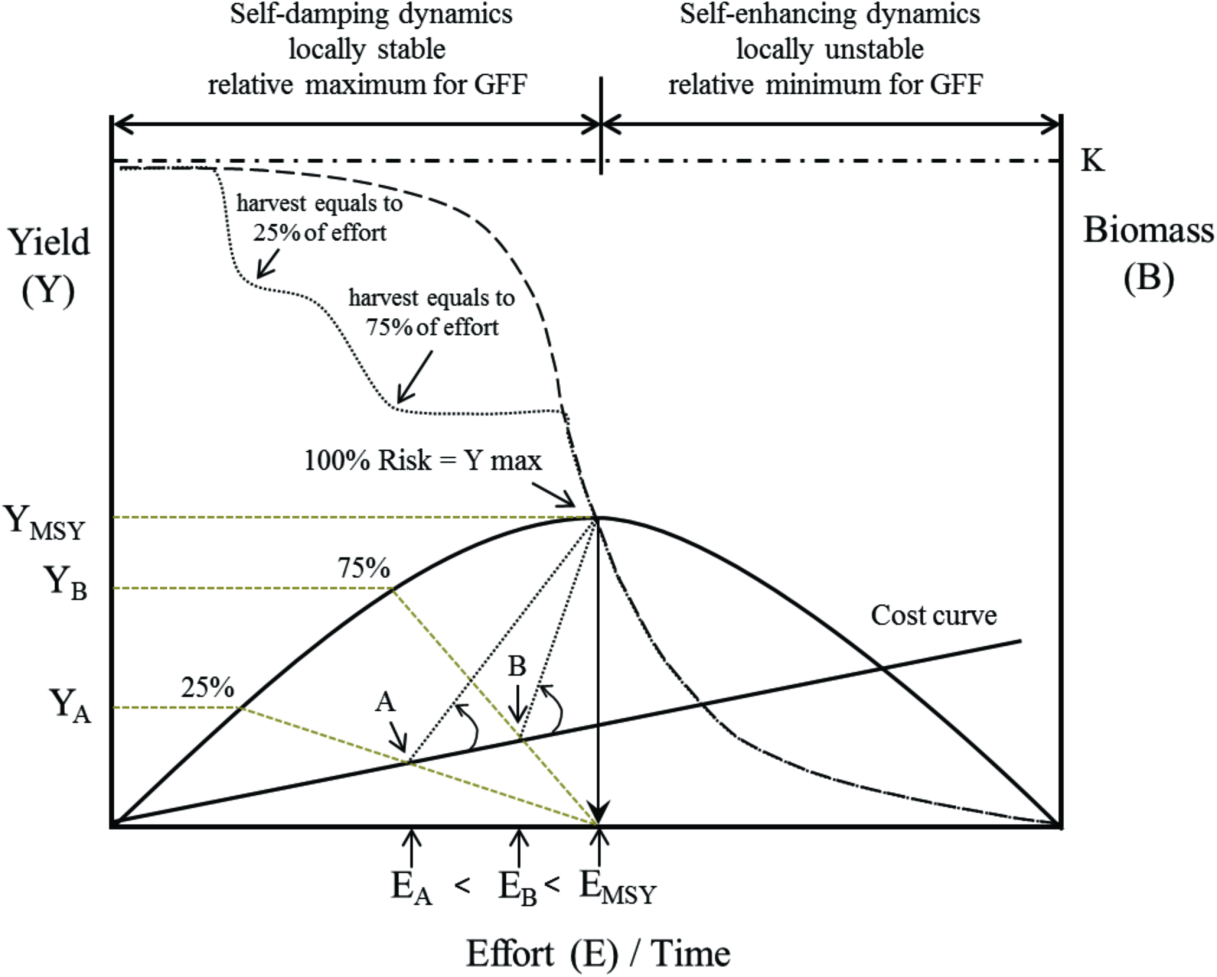
A progressive “ecological” tax for the seaweed (kelp) fishery is described. The yield curve (Y) is a quadratic curve of effort (E) with a single maximum at E_MSY_ which is reached at biomass = K/2 (K = carrying capacity) of the classic differential logistic equation. A stable relative maximum for GFF lie in the middle left of the yield curve, ensuring different intensities of harvest The progressive “ecological” tax is based on the distance to E_MSY_ (or K/2) (cero means without harvest and E_MSY_ equals maximum harvest). Points A and B represent arbitrarily different levels of taxing rates, increasing the total operational cost. For more details see text.

Based on our analysis, we propose to implement a progressive “ecological” taxing that relates the outcomes regarding local stability and relative maximum of GFF and the results obtained by the classic logistic (differential) equation. The tax proposal is described in Fig 6. The yield function, Y, is a quadratic curve of effort (E) with a single maximum at E_MSY_ which is reached at biomass = K/2 (K = carrying capacity of the logistic equation). A locally stable relative maximum for GFF lie in the middle left of the yield curve, defining the area from zero to maximum yield as the area of “population risk”, which corresponds to the maximum biomass of the resource before harvest. In this case, E_MSY_ coincides with the maximum risk for the population (this threshold separated between locally stable and unstable harvest scenarios). As a consequence, we propose a progressive tax based on the distance to E_MSY_ (or K/2). As illustrative example, the left side of the yield curve is arbitrarily divided at 25 and 75% risk (inflection points A and B, respectively), from which different levels of taxing rates are started, increasing the total artisanal fishery operational cost. As the level of landing becomes higher (approaching the biomass to K/2) the greater the tax will be which would operate similarly to a tax for discouraging the market concentration. Likewise, this progressive taxation could be implemented for any intensive artisanal fisheries with low operational cost.

This study should be considered as a general strategy for examining the consequences of human interventions in ecosystems, providing a first attempt to show -in a semi-quantitative way and under a transient dynamics- how the self-feedbacks and the success of holistic-sustainable management would depend on the positive self-feedback of the demand (economy). Likewise, the magnitude of protection (commensalism) that kelps offer to other organisms could change drastically the conclusions regarding the Hessian optimization. Finally, although one task is to determine how the sustainable exploitation of natural bio-resources and ecosystem services could solve the livelihood constrains of human beings, another different point is to perform an irrational harvest, promoting the accumulation of wealth. The approach developed herein shows the importance of combining different types of studies that tackle the interest issue from several angles, thereby providing robust conclusions.

## Supporting information

S1 Appendix Loop Analysis semi-quantitative theoretical framework and Hessian optimization procedure. (DOCX)

S2 Appendix Self-dynamics of variables. (DOCX)

S3 Appendix Qualitative Hessian optimization analysis for the eco-social models and scenarios. (DOCX)
