# Supplementary material for: Self-feedbacks determine the sustainability of human interventions in eco-social complex systems: Impacts on biodiversity and ecosystem health
Source: PLoS One. 2017 Apr 28;12(4):e0176163. doi: 10.1371/journal.pone.0176163 (PMC5409167; doi:10.1371/journal.pone.0176163)
Supplement: S1 Appendix — (DOCX) [file pone.0176163.s001.docx]

**Supplementary Information**

**Appendix S1**

- *Loop Analysis semi-quantitative theoretical framework*

Loop models show relationships as a sign, which indicates the type of influence each variable has upon another (i.e. positive, negative, or zero). For instance, in ecological relationships, (+,-) denotes a predator-prey or parasite-host interaction, (-,-) represents competition between two species, whereas (+,+), (+,0), and (-,0) represent mutualism, commensalism, and ammensalism, respectively. Each variable is represented by a node (large circle) and edges (lines) representing directions and types of interactions -an arrow at one end indicates a positive effect; a circle means the effect is negative; and the lack of a symbol shows a null effect. *Loop Analysis* is based on the correspondence between differential equations near equilibrium, matrixes and their loop diagrams. Therefore, in the benthic system, the element *a_ij_* of the matrix and the loop diagram represent the effect of variable *j* on the growth variable *i* when the equation:

$\frac{{dX}_{i}}{dt}=f_{i}(X_{1}, X_{2}, X_{3},\ldots, X_{n}; C_{1}, C_{2}, C_{3}, \ldots, C_{n}$) (1)

where the change in time of variable *X_i_* is a function *f_i_* of other interconnected variables *X_n_* and parameters *C_n_*, and is solved at equilibrium. The link from *X_j_* to *X_i_* is similar to the *α_ij_* in [1], as follows:

$\alpha_{ij}=\frac{\partial f_{i}(X)X^{*}}{\partial X_{j}}$ (2)

where *X** is evaluated at moving equilibrium. The element of the graph representing the link from *j* to *i* is sign (*α_ij_*) – whether positive, negative, or zero – where the function sign (*X*) is 1 when *X* > 0, 0 when *X* = 0, and -1 when *X* < 0.

Local stability, as determined by the Routh-Hurwitz criteria, translates into loop terms as Condition 1 when *F_k_* < 0 for all *k*; i.e. *F_k_* corresponds to the negative feedback on every level (*k*) that must exceed the positive feedback. Condition 2 indicates that negative feedback on higher levels cannot be too great compared to the negative feedback on lower levels in order to conserve the qualitative stability properties of the systems. This second condition was calculated by using the expansion of the Hurwitz determinants in terms of feedbacks or loops [2]. The feedback for each level can also be calculated by estimating the characteristic polynomial related to the Jacobean interaction matrix, in which the polynomial now can be written in terms of the feedback notation as follows:

$F_{0}\lambda^{n}+F_{1}\lambda^{n-1}+F_{2}\lambda^{n-2}+\ldots+F_{n-1}\lambda^{1}+F_{n}\lambda^{0}=0$ (3)

where *F_0_* ≡ -1 and the *F_n_* is the feedback of the entire system (*n* = total number of variables in the system) [2]. It is assumed that the system is locally stable when *F_n_* is negative. The stronger the negative feedback (*F_n_*) becomes, the greater the resistance will be to external change [3]. Based on this local stability criterion, it is possible to estimate the degree of resistance to perturbations (as a measure of sustainability) of the system and, simultaneously, to explore strategies to increase this resistance.

*Loop Analysis* [2, 3, 4] is a useful technique for estimating the local stability (sustainability) of systems and assessing the propagation of direct and indirect effects as a response to external perturbations [2]. This approach has been applied widely in different fields of the natural sciences [5, 6, 7] including fisheries management [8, 9, 10, 11, 12, 13, 14] and malaria control interventions [15], and has shown a high degree of predictability (after perturbation) of natural phenomena [16, 17, 18, 19, 20, 21].

- *Hessian optimization procedure*

The Hessian optimization analysis considers a multidimensional function as follows:

$\frac{dH}{dt}=f(X_{1}, X_{2}, X_{3}, \ldots, X_{n})$ (4)

where *H* is the multidimensional function varying continuously in an open region, and *X_i_* the variables. Consider the set of determinants ǀ *D_i_* ǀ, *i* = 1, 2, 3, …., *n*, where

$\left| D_{i} \right|=\left| \begin{matrix} \frac{\partial^{2}H}{\partial X_{1}^{2}} & \frac{\partial^{2}H}{\partial X_{1}\partial X_{2}} & . & . & . & \frac{\partial^{2}H}{\partial X_{1}\partial X_{i}} \\ \frac{\partial^{2}H}{\partial X_{2}\partial X_{1}} & \frac{\partial^{2}H}{\partial X_{2}^{2}} & . & . & . & \frac{\partial^{2}H}{\partial X_{2}\partial X_{i}} \\ . & . & . & . & . & . \\ . & . & . & . & . & . \\ . & . & . & . & . & . \\ \frac{\partial^{2}H}{\partial X_{i}\partial X_{1}} & \frac{\partial^{2}H}{\partial X_{i}\partial X_{2}} & . & . & . & \frac{\partial^{2}H}{\partial X_{i}^{2}} \end{matrix} \right|$ (5)

If *∂H/∂X_1_* = *∂H/∂X_2_* = *∂H/∂X_n_* = 0 at (*X_10_*, *X_20_*, …, *X_n0_*), then it is possible to establish the following conditions of optimization:

1.- ǀ *D_i_* ǀ < 0 for *i* = 1, 3, 5, … , and ǀ *D_i_* ǀ > 0 for *i* = 2, 4, 6, … indicates the presence of a relative maximum at *X_10_*, *X_20_*, …., *X_n0_*.

2.- ǀ *D_i_* ǀ > 0 for *i* = 1, 2, 3, …, *n* indicates the presence of a relative minimum at *X_10_*, *X_20_*, …., *X_n0_*.

Any departure from these conditions corresponds to a saddle-point, that is, without maximum neither minimum [22].

**References**

1. Levins R (1968) Evolution in changing environments. Princeton Monographs Series, Princeton.

2. Puccia Ch, Levins R (1985) Qualitative modeling of complex systems: an introduction to Loop Analysis and time averaging. Harvard University Press, Cambridge, MA.

3. Levins R (1998) Qualitative mathematics for understanding, prediction, and interventions in complex ecosystems. In: Rapport D, Costanza R, Epstein P, Gaudet C, Levins R, editors. Ecosystem Health. Blackwell Science, Oxford, pp. 178-204.

4. Levins R (1974) The qualitative analysis of partially specified systems. Annals of the New York Academy of Sciences 231: 123-138.

5. Ramsey D, Veltman C (2005) Predicting the effects of perturbations on ecological communities: what can qualitative models offer? Journal of Animal Ecology 74: 905-916.

6. Li H, Moyle P (1981) Ecological analysis of species introductions into aquatic systems. Transactions of the American Fisheries Society 110: 772-782.

7. Giavelli G, Bodini A (1990) Plant-ant-fungus communities investigated through qualitative modeling. Oikos 57: 357-365.

8. Ortiz M, Levins R (2011) Re-stocking practices and illegal fishing in northern Chile (SE Pacific coast): a study case. Oikos 120: 1402-1412.

9. Ortiz M (2003) Qualitative modelling of the kelp forest of *Lessonia nigrescens* Bory (Laminariales: Phaeophyta) in eulittoral marine ecosystems of the south-east Pacific: an approach to management plan assessment. Aquaculture 220: 423-436.

10. Lane P (1998) Assessing cumulative health effects in ecosystems. In: Rapport D, Costanza R, Gaudet C, Levins R, editors. Ecosystem Health. Blackwell Science, Oxford, pp. 129-153.

11. Ortiz M, Wolff M (2002) Application of loop analysis to benthic systems in northern Chile for the elaboration of sustainable management strategies. Marine Ecology Progress Series 242: 15-2.

12. Ortiz M, Stotz W (2007) Ecological and eco-social analysis for the introduction of the abalon *Haliotis discus hannoi* into benthic systems of central-north Chile. Aquatic Conservation: Marine and Freshwater Ecosystems 17: 89-105.

13. Dambacher J, Guaghan D, Rochet M, Rossignol P, Trenkel V (2009) Qualitative modeling and indicators of exploited ecosystems. Fish and Fisheries 10: 305-322.

14. Ortiz M, Rodríguez-Zaragoza F, Hermosillo-Núñez B, Jordán F (2015) Control strategy scenarios for the alien lionfish *Pterois volitans* in Chinchorro Bank (Mexican Caribbean): based on semi-quantitative Loop Analysis. PLoS ONE 10(6): e0130261.doi:10.1371/journal.pone.0130261.

15. Reum J, McDonald P, Ferris B, Farrell D, Harvey C, Levin P (2015) Qualitative network models in support of ecosystem approaches to bivalve aquaculture. ICES Journal of Marine Sciences 78(8): 2278-2288.

16. Yasouka J, Jimba M, Levins R (2014) Application of loop analysis for the evaluation of malaria control) interventions. Malaria Journal 13: 1-15.

17. Briand F, McCauley E (1978) Cybernetic mechanisms in lake plankton systems: how to control undesirable algae. Nature 273: 228-230.

18. Lane P, Collins T (1985) Food web models of a marine plankton community network: an experimental mesocosm approach. Journal of Experimental Marine Biology and Ecology 94: 41-70.

19. Lane P (1986) Symmetry, change, perturbation, and observing mode in natural communities. Ecology 67(1): 223-239.

20. Hulot F, Lacroix G, Lescher-Moutoué F, Loreau M (2000) Functional diversity governs ecosystem response to nutrient enrichment. Nature 405: 340-344.

21. Ortiz M (2008) The effect of a crab predator (*Cancer porteri*) in secondary producers versus ecological model predictions in Tongoy Bay (south-east Pacific coast): implications for management and fisheries. Aquatic Conservation: Marine and Freshwater Ecosystems 18: 923-929.

22. Gottfried B, Weisman J (1973) Introduction to optimization theory. Englewoods Cliffs, Prentice –Hall.
