# Supplementary material for: Self-feedbacks determine the sustainability of human interventions in eco-social complex systems: Impacts on biodiversity and ecosystem health
Source: PLoS One. 2017 Apr 28;12(4):e0176163. doi: 10.1371/journal.pone.0176163 (PMC5409167; doi:10.1371/journal.pone.0176163)
Supplement: S2 Appendix — (DOCX) [file pone.0176163.s002.docx]

**Supplementary Information**

**Appendix S2**

- *Self-dynamics of the variables*

Eco-social Model 1

This model includes only two variables: fishers (*F*) and the group of seaweeds or kelp species (*X*). Both variables are connected by a prey-predator relationship. The equations for each variable are the following:

*dX/dt* = *r_x_X – aXF ± bX^2^* (1)

*dF/dt = q_x_E_x_X – wLF ± ΦF^2^* (2)

The self-enhancing and self-damping dynamics of *X* (kelps) is obtained as follows:

*∂(dX/dt)/∂X* = *r_x_* – *aF* ± *2bX* (3)

solving at equilibrium we obtain that self-enhancing is *X** = (*aF* - *r_x_*) / *2b*, and the self-damping corresponds to *X** = (*r_x_* – *aF*) / *2b*.

Both self-dynamics of *F* are obtained as follows:

*∂(dF/dt)/∂F* = – *wL* ± *2 ΦF* (4)

solving also at equilibrium we have *F** = *wL* / *2Φ*, and *F** = - *wL* / *2Φ* for self-enhancing and self-damping respectively.

Eco-social Model 2

The eco-social Model 2 includes one new variable, the demand (*D*). The equations for each variable are:

*dX/dt* = *r_x_X – aXF ± bX^2^* (5)

*dF/dt = q_x_E_x_X + cDF – wLF ± ΦF^2^* (6)

*dD/dt* = *αX^2^* – *dFD* + *eD^2^* (7)

The self-enhancing and self-damping dynamics of *X* (kelps) is similar to eco-social Model 1.

Both self-dynamics of *F* are obtained as follows:

*∂(dF/dt)/∂F* = *cD* – *wL* ± *2ΦF* (9)

solving also at equilibrium we have *F** = (*wL* – *cD*) / *2Φ*, and *F** = (*cD* – *wL*) / *2Φ* for self-enhancing and self-damping respectively.

In the case of demand (*D*), the self-enhancing dynamics is obtained as follows:

*∂(dD/dt)/∂D* = - *dF* + *2eD* (10)

solving at equilibrium we obtain *D** = *dF* / *2e*.

Eco-social Model 3

This includes one new variable corresponding to the keyhole limpet group (*Y*), which is connected with the seaweeds by a plant-herbivore relationship. The equation for each variable corresponds to:

*dX/dt* = *r_x_X - aXF* - *μXY ± bX^2^* (11)

*dY/dt = μ_1_YX - gYF ± hY^2^* (12)

*dF/dt = q_x_E_x_X +q_y_E_y_Y +cDF – wLF ± ΦF^2^* (13)

*dD/dt* = *αX^2^* + *βY^2^*– *dFD* + *eD^2^* (14)

The self-dynamics of kelps (*X*) is obtained as follows:

*∂(dX/dt)/∂X* = *r_x_ - aF* - *μY ± 2bX* (15)

solving at equilibrium we obtain that self-enhancing is *X** = (*aF* + *μY* - *r_x_*) / *2b*, and the self-damping corresponds to *X** = (*r_x_* – *aF* - *μY*) / *2b*.

The self-dynamics of limpets (*Y*) is estimated as follows:

*∂(dY/dt)/∂Y* = *μ_1_X - gF ± 2hY.* (16)

solving at equilibrium, the positive self-feedbacks for *Y** = (*gF* - *μ_1_X*) / *2h*, and the negative is *Y** = (*μ_1_X – gF*) / *2h.*

Both self-dynamics of *F* and the positive dynamics of *D* are similar to the eco-social Model 2.

Eco-social Model 4

This model incorporates one new variable the crab group (*Z*), which is connected as a predator upon keyhole limpets and being positively impacted by the seaweeds (commensalism). The equation for each variable is:

*dX/dt* = *r_x_X - aXF* - *μXY ± bX^2^* (17)

*dY/dt = μ_1_YX - θYZ - gYF ± hY^2^* (18)

*dZ/dt* = *θ_1_ZY +λX^2^ – mZF* *± nZ^2^*  (19)**

*dF/dt = q_x_E_x_X +q_y_E_y_Y +q_z_E_z_Z* + *cDF – wLF ± ΦF^2^* (20)

*dD/dt* = *αX^2^* + *βY^2^* + *δZ^2^* – *dFD* + *eD^2^* (21)

The self-dynamics of kelps (*X*) is similar to eco-social Model 3.

The self-dynamics of limpets (*Y*) is estimated as follows:

*∂(dY/dt)/∂Y* = *μ_1_X - θZ - gF ± 2hY* (22)

solving at equilibrium, the positive self-feedbacks for *Y** = (*θZ* + *gF* - *μ_1_X*) / *2h*, and the negative is *Y** = (*μ_1_X – θZ - gF*) / *2h.*

The self-enhancing and self-damping dynamics of crabs (*Z*) is obtained as follows:

*∂(dZ/dt)/∂Z* = *θ_1_Y – mF* *± 2 nZ* (23)

solving at equilibrium, the self-positive feedbacks for *Z** = (*mF* - *θ_1_Y*) / *2n*, and the negative is *Z** = (*θ_1_Y* - *mF*) / *2n*.

Both self-dynamics of *F* and the positive dynamics of *D* are similar to the eco-social Model 2.
