# Supplementary material for: Self-feedbacks determine the sustainability of human interventions in eco-social complex systems: Impacts on biodiversity and ecosystem health
Source: PLoS One. 2017 Apr 28;12(4):e0176163. doi: 10.1371/journal.pone.0176163 (PMC5409167; doi:10.1371/journal.pone.0176163)
Supplement: S3 Appendix — (DOCX) [file pone.0176163.s003.docx]

**Supplementary Information**

**Appendix S3**

- *Qualitative Hessian optimization analysis for the eco-social models and scenarios.*

**QUALITATIVE ANALYSIS**

**ECO-SOCIAL MODEL 1: Hessian matrix**

|  | ***X*** | ***F*** |
| --- | --- | --- |
| ***X*** | ∂^2^GFF/∂X^2^ = ±2b | ∂^2^GFF/∂X∂F = -a |
| ***F*** | ∂^2^GFF/∂F∂X = -a | ∂^2^GFF/∂F^2^ = ±2φ |

**Four conditions:**

**(1) MEABRs scenario: *X* and *Y* are under self-damping dynamics.**

D_i=1_ = < 0

D_i=2_ = + 4bφ - a^2^, if the self-damping is stronger than other constants; > 0

* Therefore, a relative maximum for *GFF* is obtained.

These results seem to be expected, since if the abundance of species *X* (resource) is near to their carrying capacity (*K,* of logistic equation) then the abundance is the highest and fishing activity can be maximized. Contrary, if the abundance is below *K/2*, then the harvest could be minimized. In the case of abundance of *X* = *K/2* a saddle-point is found.

**(2) OAAs scenario: X and Y are under self-enhancing dynamics.**

D_i=1_ = > 0

D_i=2_ = + 4bφ - a^2^, if the self-enhancing is stronger than other constants; > 0

* Therefore, a relative minimum for *GFF* is found.

**ECO-SOCIAL MODEL 2: Hessian matrix**

|  | ***X*** | ***F*** | ***D*** |
| --- | --- | --- | --- |
| ***X*** | ±2b + 2α | -a | 0 |
| ***F*** | -a | ±2φ | c - d |
| ***D*** | 0 | c - d | +2e |

**Additional analysis**

**(1) MEABRs scenario: *X* and *F* are self-damping and D is self-enhancing.**

Assumptions: c ≠ d; b >> α; -2b + 2α = -P

D_i=1_ = < 0

D_i=2_ = + 2Pφ - a^2^, > 0

D_i=3_ = + 4Pφe + Pc^2^ + Pd^2^ – 2Pcd - 2a^2^e, > 0

* Therefore, a saddle-point for *GFF* is found.

**(2) OAAs scenario: the three variables are under self-enhancing dynamics.**

Assumptions: c ≠ d; b >> α; 2b + 2α = L

D_i=1_ = > 0

D_i=2_ = + 2Lφ - a^2^, > 0

D_i=3_ = + 4Lφe + 2Lcd – Lc^2^ – Ld^2^ - 2a^2^e, > 0

* Therefore, a relative minimum for *GFF* is reached.

**(3) Sustainable scenario: the three variables are under self-damping dynamics.**

Assumptions: c ≠ d; b >> α; -2b + 2α = -P

D_i=1_ = < 0

D_i=2_ = + 8Pφ - a^2^, > 0

D_i=3_ = - 4Pφe – 2Pcd + Pc^2^ + Pd^2^ + 2a^2^e, < 0

* Therefore, a relative maximum for *GFF* is found.

**ECO-SOCIAL MODEL 3: Hessian matrix**

|  | ***X*** | ***Y*** | ***F*** | ***D*** |
| --- | --- | --- | --- | --- |
| ***X*** | ±2b + 2α | -μ + μ_1_ | -a | 0 |
| ***Y*** | -μ + μ_1_ | ±2h + 2β | -g | 0 |
| ***F*** | -a | -g | ±2φ | c - d |
| ***D*** | 0 | 0 | c - d | +2e |

**Additional analysis**

**(1) MEABRs scenario: *X*, *Y* and *F* are self-damping and *D* is self-enhancing.**

Assumptions: c ≠ d; -μ ≠ μ_1_; b >> α; h >> β; -2b + 2α = -P; -2h + 2β = -T.

D_i=1_ = < 0

D_i=2_ = + PT + 2µµ_1_ - µ^2^ -µ_1_^2^, > 0

D_i=3_ = - PTφ - µga - 4µµ_1_φ - abµ + Pbg + Ta^2^ + 2φµ^2^ + 2φµ_1_^2^ + gaµ_1_ + abµ_1_, < 0

D_i=4_ = - 4PTφe – PTc^2^ – PTd^2^ – 2cdµ^2^ - 8µµ_1_φe - 2µµ_1_c^2^ - 2µµ_1_d^2^ - 4µgae – 2µ^2^cd - 2µ_1_^2^cd + 2PTcd + 2Pg^2^e + 2Ta^2^e + 4µ^2^φe + µ^2^c^2^ + µ^2^d^2^ + 4µµ_1_cd + 4µ_1_^2^φe + µ_1_^2^c2 + µ_1_^2^d^2^ + 4µ_1_gae, < 0

* Therefore, a saddle-point for *GFF* is found.

**(2) OAAs scenario: the four variables are under self-enhancing dynamics.**

Assumptions: c ≠ d; -μ ≠ μ_1_; b >> α; h >> β; 2b + 2α = L; 2h + 2β = S.

D_i=1_ = > 0

D_i=2_ = + LS + 2µµ_1_ - µ^2^ - µ_1_^2^, > 0

D_i=3_ = + 2LSφ + 4µµ_1_φ + 2µ_1_ga – Lg^2^ – Sa^2^ - 2µga - 2φµ^2^ - 2φµ_1_^2^, > 0

D_i=4_ = + 4LSφe + 2LScd + 4µ_1_gae + 4µµ_1_cd + 8µµ_1_φe + µ^2^c^2^ + µ^2^d^2^ + µ_1_^2^c^2^ + µ_1_^2^d^2^ – LSc^2^ – LSd^2^ – 2Lg^2^e – 2Sa^2^e - 4µ^2^φe - 4µgae - 4µ_1_^2^φe - 2µµ_1_c^2^ - 2µµ_1_d^2^ - 2µ^2^cd - 2µ_1_^2^cd, > 0

* Therefore, a relative minimum for *GFF* is reached.

**(3) Sustainable scenario: the four variables are under self-damping dynamics.**

Assumptions: c ≠ d; -μ ≠ μ_1_; b >> α; h >> β; -2b + 2α = -P; -2h + 2β = -T.

D_i=1_ = < 0

D_i=2_ = + PT + 2µµ_1_ - µ^2^ - µ_1_^2^, > 0

D_i=3_ = - 2PTφ - 2µga - 4µµ_1_φ + Pg^2^ + Ta^2^ + 2µ_1_ga + 2φµ^2^ + φµ_1_^2^, < 0

D_i=4_ = + 4PTφe + 2PTcd + 8µµ_1_φe + 4µgae + 4µµ_1_cd + µ^2^c^2^ + µ^2^d^2^ + µ_1_^2^c^2^ + µ_1_^2^d^2^ – PTc^2^ – PTd^2^ – 2Pg^2^e – 2Ta^2^e - 4µ^2^φe - 4µ_1_^2^φe - 4µ_1_gae - 2µµ_1_c^2^ - 2µµ_1_d^2^ - 2µ^2^cd - 2µ_1_^2^cd, > 0

* Therefore, a relative maximum for *GFF* is found.

**ECO-SOCIAL MODEL 4: Hessian matrix**

|  | ***X*** | ***Y*** | ***Z*** | ***F*** | ***D*** |
| --- | --- | --- | --- | --- | --- |
| ***X*** | ±2b + 2α + 2λ | -μ + μ_1_ | 0 | -a | 0 |
| ***Y*** | -μ + μ_1_ | ±2h + 2β | -θ + θ_1_ | -g | 0 |
| ***Z*** | 0 | -θ + θ_1_ | ±2n + 2δ | -m | 0 |
| ***F*** | -a | -g | -m | ±2φ | c – d |
| ***D*** | 0 | 0 | 0 | c – d | +2e |

**Additional analysis**

1. **MEABRs scenario: *X*, *Y*, *Z* and *F* are self-damping and *D* is self-enhancing.**

Assumptions: c ≠ d; -μ ≠ μ_1_; -θ ≠ θ_1_; b >> α; b >> λ; h >> β; n >> δ; -2b + 2α +2λ = -P; -2h + 2β = -T; -2n + 2δ = -U.

D_i=1_ = < 0

D_i=2_ = + PT + 2µµ_1_ - µ^2^ - µ_1_^2^, > 0

D_i=3_ = - PTU – 2Pθθ_1_ – 2Uµµ_1_ + Pθ^2^ + Pθ_1_^2^ + Uµ^2^ + Uµ_1_^2^, < 0

D_i=4_ = + 2PTUφ + 4Pφθθ_1_ + 2Pθmg + 2Uµga + 4Uφµµ_1_ + 2µθ_1_ma + 2µ_1_θma + µ^2^m^2^ - µ_1_^2^m^2^ + a^2^θ^2^ + a^2^θ_1_^2^ – PTm^2^ – Pug^2^ – Tua^2^ – 2Pφθ^2^ – 2Pφθ_1_^2^ – 2Pθ_1_mg – 2Uφµ^2^ – 2Uφµ_1_^2^ – 2Uµ_1_ga – 2µθma – 2µ_1_θ_1_ma – 2µµ_1_m^2^ – 2ª^2^θθ_1_, > 0

D_i=5_ = + 4PTUφe + PTUc^2^ + PTUd^2^ + 8Pφeθθ_1_ + 4Pθmge + 8Uφeµµ_1_ + 4Ueµga + 2Pθθ_1_c^2^ + 2Pθθ_1_d^2^ + 2Pθ_1_^2^cd + 2Pθ^2^cd + 2Uµ^2^cd + 2Uµ_1_^2^cd + 2Uµµ_1_c^2^ + 2Uµµ_1_d^2^ + 4µθ_1_mae + 4µ_1_θmae + 2µ^2^m^2^e + 2ª^2^θ_1_^2^e + 2ª^2^θ^2^e + 2µ_1_^2^m^2^e – 2PTUcd – 2PTm^2^e -2Pug^2^e – 2Tua^2^e – 4Pφθ^2^e – 4Pφθ_1_^2^e – 4Pθ_1_mge – 4Pθθ_1_cd – 4Uµ_1_gae – 4Uµ^2^φe – 4Uµ_1_^2^φe – 4Uµµ_1_cd – 4µµ_1_m^2^e – 4θθ_1_ª^2^e – 4µθmae – 4µ_1_θ_1_mae – Pθ^2^d^2^ – Pθ_1_^2^d^2^ – Pθ^2^c^2^ – Pθ_1_^2^c^2^ – Uµ^2^d^2^ – Uµ^2^c^2^ – Uµ_1_^2^d^2^ – Uµ_1_^2^c^2^, > 0

* Therefore, a saddle-point for *GFF* is found.

**(2) OAAs scenario: the five variables are under self-enhancing dynamics.**

Assumptions: c ≠ d; -μ ≠ μ_1_; -θ ≠ θ_1_; b >> α; b >> λ; h >> β; 2b + 2α + 2λ = L; 2h + 2β = S; 2n + 2δ = V.

D_i=1_ = > 0

D_i=2_ = + LS + 2µµ_1_ - µ^2^ - µ_1_^2^, > 0

D_i=3_ = + LSV + 2Lθθ_1_ + 2Vµµ_1_ – Lθ^2^ – Lθ_1_^2^ – Vµ^2^ – Vµ_1_^2^, > 0

D_i=4_ = + 2LSVφ + 4Lφθθ_1_ + 4Vφµµ_1_ + 2Lθ_1_mg + 2Vµ_1_ga + 2µθ_1_ma + 2µ_1_θma + µ^2^m^2^ + µ_1_^2^m^2^ + a^2^θ^2^ + a^2^θ_1_^2^ – LSm^2^ – LVg^2^ – Sva^2^ – 2Lφθ^2^ – 2Lφθ_1_^2^ – 2Vφµ^2^ – 2Vφµ_1_^2^ – 2Lθmg – 2Vµga – 2µθma – 2µ_1_θ_1_ma – 2µµ_1_m^2^ – 2ª^2^θθ_1_, > 0

D_i=5_ = + 4LSVφe + 2LSVcd + 8Lφeθθ_1_ + 4Lθ_1_mge + 4Lθθ_1_cd + 8Vφeµµ_1_ + 4Vµµ_1_cd + 4Vµ_1_gae + Lθ_1_^2^d^2^ + Lθ^2^d^2^ + Lθ_1_^2^c^2^ + Lθ^2^c^2^ + Vµ^2^d^2^ + Vµ_1_^2^d^2^ + Vµ^2^c^2^ + Vµ_1_^2^c^2^ + 4µ_1_θmae + 4µθ_1_mae + 2µ^2^m^2^e + 2µ_1_^2^m^2^e + 2ª^2^θ^2^e + 2 a^2^θ_1_^2^e – LSVd^2^ – LSVc^2^ -2LSm^2^e – 2LVg^2^e – 2Sva^2^e – 4Lφθ_1_^2^e – 4Lφθ^2^e – 4Vµ^2^φe – 4Vµ_1_^2^φe – 4Veµga – 4Lθmge – 2Lθθ_1_c^2^ – 2Lθθ_1_d^2^ – 2Lθ^2^cd – 2Lθ_1_^2^cd – 2Vµ^2^cd – 2Vµ_1_^2^cd – 2Vµµ_1_c^2^ – 2V µµ_1_d^2^ – 4µµ_1_m^2^e – 4θθ_1_ª^2^e – 4µθmae – 4µ_1_θ_1_mae, > 0

* Therefore, a relative minimum for *GFF* is reached.

**(3) Sustainable scenario: the five variables are under self-damping dynamics.**

Assumptions: c ≠ d; -μ ≠ μ_1_; -θ ≠ θ_1_; b >> α; b >> λ; h >> β; n >> δ; -2b + 2α +2λ = -P; -2h + 2β = -T; -2n + 2δ = -U.

D_i=1_ = < 0

D_i=2_ = + PT + 2µµ_1_ - µ^2^ - µ_1_^2^, > 0

D_i=3_ = - PTU – 2Pθθ_1_ – 2Uµµ_1_ + Pθ^2^ + Pθ_1_^2^ + Uµ^2^ + Uµ_1_^2^, < 0

D_i=4_ = + 2LSVφ + 4Lφθθ_1_ + 4Vφµµ_1_ + 2Lθ_1_mg + 2Vµ_1_ga + 2µθ_1_ma + 2µ_1_θma + µ^2^m^2^ + µ_1_^2^m^2^ + a^2^θ^2^ + a^2^θ_1_^2^ – LSm^2^ – LVg^2^ – Sva^2^ – 2Lφθ^2^ – 2Lφθ_1_^2^ – 2Vφµ^2^ – 2Vφµ_1_^2^ – 2Lθmg – 2Vµga – 2µθma – 2µ_1_θ_1_ma – 2µµ_1_m^2^ – 2ª^2^θθ_1_, > 0

D_i=5_ = - 4PTUφe – 2PTUcd – 8Pφeθθ_1_ – 8Uφeµµ_1_ – 4Pθmge – 4Ueµga – 4Pθθ_1_cd – 4Uµµ_1_cd – Pθ^2^c^2^ – Pθ^2^d^2^ – Pθ_1_^2^d^2^ – Pθ_1_^2^c^2^ – Uµ^2^c^2^ – Uµ_1_^2^c^2^ – Uµ^2^d^2^ – Uµ_1_^2^d^2^ – 4µ_1_θmae – 4 µθ_1_mae – 2ª^2^θ^2^e – 2ª^2^θ_1_^2^e – 2µ^2^m^2^e – 2 µ_1_^2^m^2^e + PTUc^2^ + PTUd^2^ + 2PTm^2^e + 2Pug^2^e + 2Tua^2^e + 4Pθ_1_mge + 4Pφθ_1_^2^e + 4Pφθ^2^e + 4Uµ^2^φe + 4Uµ_1_^2^φe + 2Pθ^2^cd + 2Pθ_1_^2^cd + 2Pθθ_1_c^2^ + 2Pθθ_1_d^2^ + 4Uµ_1_gae + 2U µµ_1_c^2^ + 2U µµ_1_d^2^ + 2Uµ^2^cd + 2Uµ_1_^2^cd + 4µµ_1_m^2^e + 4θθ_1_ª^2^e + 4µθmae + 4 µ_1_θ_1_mae, < 0

* Therefore, a relative maximum for *GFF* is found.
